# Anti-ethylene Diamine Tetra-Acetate (EDTA)-Induced Pseudothrombocytopenia in a Nigerian Middle-Aged Man

**DOI:** 10.7759/cureus.82864

**Published:** 2025-04-23

**Authors:** Pelumi V Ajayi, Olatokunbo O Oseni, Bamikole Osibowale, Ekido R Okpiabhele, Olurotimi J Badero, Victor O Adedara

**Affiliations:** 1 General Medicine, Iwosan Lagoon Hospital, Lagos, NGA; 2 Family Medicine, Iwosan Lagoon Hospitals, Lagos, NGA; 3 Cardiology, Tristate Heart Institute, Lagos, NGA; 4 Internal Medicine, Iwosan Lagoon Hospitals, Lagos, NGA; 5 Interventional Cardiology, Iwosan Lagoon Hospital, Lagos, NGA; 6 Interventional Cardiology, Division of Cardio-Nephrology, Cardiac Renal & Vascular Associates, Jackson, USA; 7 Medicine, St. George's University School of Medicine, West Indies, GRD

**Keywords:** anti-edta, blood, hematology, platelet glycoproteins (gp) iib/iiia, pseudothrombocytopenia

## Abstract

Ethylene diamine tetra-acetate-induced pseudothrombocytopenia (EDTA-PTCP) is a rare laboratory phenomenon where antibodies target platelet glycoproteins (GP) IIb/IIIa, causing platelet clumping and falsely low platelet counts. This can lead to misdiagnosis, unnecessary treatments, and an artificially elevated white blood cell count.

We report a 51-year-old man referred for worsening thrombocytopenia without signs of active bleeding. He had persistent low-grade fever and generalized weakness while receiving treatment for malaria. At our facility, his platelet count dropped from 25,000/mm³ to 9,500/mm³. Suspecting EDTA-PTCP, we requested a peripheral blood film and repeated testing using a different anticoagulant. The results confirmed platelet clumping, revealing that the initial thrombocytopenia was spurious.

EDTA-PTCP may be underrecognized, leading to unnecessary interventions. Awareness and early suspicion are essential to avoid misdiagnosis and ensure accurate patient management.

## Introduction

While ethylene diamine tetra-acetate (EDTA) is the anticoagulant of choice in routine hematological testing due to its ability to preserve cellular components, its use is not without pitfalls. One such limitation is EDTA-induced pseudothrombocytopenia (EDTA-PTCP), a rare but clinically significant phenomenon that can lead to diagnostic confusion. To comprehend how EDTA-PTCP can complicate clinical interpretation, it is essential to understand the role of various anticoagulants in laboratory testing. Sodium citrate, for instance, is commonly used for coagulation studies and red cell fragility tests, while lithium heparin is favored in specialized chemistry assays.

Although EDTA is widely used for blood cell counts due to its ability to maintain cellular morphology, it can sometimes cause platelet aggregation, leading to falsely low platelet counts when analyzed by automated hematology analyzers [1,2]. This phenomenon, known as EDTA-PTCP, was first described by Gowland et al. in 1969 [3].

While EDTA-PTCP is generally harmless, it can result in misdiagnosis, unnecessary investigations, and costly interventions. The spurious low platelet count caused by EDTA can be avoided using alternative anticoagulants, such as sodium citrate or lithium heparin. In some instances, collecting blood at 37°C or using aminoglycoside-supplemented anticoagulants may also help prevent platelet aggregation [4].

## Case presentation

A 51-year-old man was referred to our facility due to a persistent decline in platelet levels. His symptoms began seven days before presentation with generalized body aches and a low-grade fever. He initially sought care at a peripheral hospital, where a thick film malaria parasite test was positive. A full blood count (FBC) showed a white blood cell (WBC) count of 3,910/mm³ (normal: 4,000-11,000/mm³), neutrophil percentage of 74.7%, platelet count of 155,000/mm³ (normal: 150,000-400,000/mm³), hemoglobin (Hb) of 12.7 g/dL (normal: 12.5-14.5 g/dL), and hematocrit of 41.3% (normal: 40%-48%). Based on these findings, he was started on oral artemisinin combination therapy (ACT) and oral amoxicillin-clavulanic acid. Given his clinical presentation and recent treatment, potential diagnoses considered included severe thrombocytopenia associated with malaria and drug-induced thrombocytopenia.

He developed a generalized body rash and hives following oral antibiotics, which resolved with oral antihistamines. However, symptoms recur upon re-exposure to the antibiotic, leading to its discontinuation. Despite completing the anti-malarial treatment, his symptoms persisted. A repeat blood test showed a drop in platelet count from 155,000/mm³ to 77,000/mm³, while the thick film malaria parasite test remained positive. As a result, he was started on intramuscular Arteether (150 mg daily for three days).

A follow-up FBC revealed a further decline in platelet count to 13,000/mm³, prompting his referral to our facility for expert evaluation. There was no history of hemoptysis, hematemesis, hematochezia, or melena. Additionally, he had no history of bleeding under the skin or from craniofacial orifices and no known family history of bleeding disorders.

On admission, a repeat FBC and malaria parasite test revealed a platelet count of 25,000/mm³ and a still-positive malaria parasite test. The patient was started on intravenous Artesunate for 24 hours. A repeat FBC and microscopic peripheral blood film examination was conducted 24 hours after admission (Table 1).

**Table 1:** Hematological parameters on admission ↑ = Above reference range; ↓ = Below reference range.

Lab test	Result	Reference range
Hemoglobin (Hb)	12.4 g/dL	12.0 – 16.0 g/dL
Packed cell volume (PCV)	39.10%	30.0 – 48.0%
White blood cell (WBC) count	6.5 cells/L	4.0 – 11.0 cells/L
Neutrophils	55.80%	40.0 – 70.0%
Lymphocytes	27.70%	25.0 – 50.0%
Monocytes	12.4% ↑	2.0 – 10.0%
Eosinophils	3.90%	0.0 – 7.0%
Basophils	0.30%	0.0 – 1.0%
Platelet count	25.8 cells/L ↓	150 – 400 cells/L
Red blood cell (RBC) count	3.7/µL ↓	4.00 – 6.20/µL
Mean corpuscular volume (MCV)	104.8 fL ↑	76 – 96 fL
Mean corpuscular hemoglobin (MCH)	33.2 pg ↑	27.0 – 32.0 pg
Mean corpuscular hemoglobin concentration (MCHC)	31.7 g/dL	31.0 – 36.0 g/dL

Suspecting drug-associated thrombocytopenia and wanting to rule out pseudo-thrombocytopenia, platelet counts were compared using blood samples collected in different anticoagulants. The platelet count in an EDTA sample was 9,500/mm³, while sodium citrate and lithium heparin samples showed 151,900/mm³ and 148,500/mm³, respectively (Table 2). Microscopic examination of the EDTA sample revealed clumped, scanty giant platelets, confirming the diagnosis of pseudo-thrombocytopenia secondary to anti-EDTA antibodies.

**Table 2:** Comparison of hematological parameters in blood samples collected with different anticoagulants (sodium citrate, lithium heparin, and EDTA) PCV: Packed cell volume, WBC: white blood cell, RBC: red blood cell, MCV: mean corpuscular volume, MCH: mean corpuscular hemoglobin, MCHC: mean corpuscular hemoglobin concentration, EDTA: Ethylene diamine tetra-acetate. Significant deviations from normal reference ranges are indicated by upward (↑) or downward (↓) arrows.

Lab test	Sodium citrate	Lithium heparin	EDTA	Reference range
Hemoglobin (g/dL)	10.5	12.3	12.29	12.0 – 16.0 g/dL
PCV (%)	32.7	38.4	38.3	30.0 – 48.0%
WBC (cells/L)	4.14 ×10⁹	4.8 ×10⁹	6.39 ×10⁹	4.0 – 11.0 ×10⁹/L
Neutrophils (%)	49	49	47.13	40 – 70%
Lymphocyte (%)	35	36	34.39	25 – 50%
Monocyte (%)	7	7	11.19 ↑	2 – 10%
Eosinophils (%)	3	3	7.13 ↑	0 – 7%
Basophils (%)	0.5	0.5	0.19 ↓	0 – 1%
Platelet (×10⁹/L)	151.9	148.5	9.5 ↓	150 – 400 ×10⁹/L
RBC (/µL)	4.2	4.4	3.66 ↓	4.00 – 6.20 ×10¹²/L
MCV (fL)	85	87	104.7 ↑	76 – 96 fL
MCH (pg)	29	30	33.6 ↑	27.0 – 32.0 pg
MCHC (g/dL)	33	34	32.1	31.0 – 36.0 g/dL

## Discussion

The case presentation figure reveals a significant variation in platelet counts among different anticoagulant samples. The EDTA sample exhibits a notably low platelet count of 9.5 ×10^9^/L, starkly contrasting 151.9 ×10^9^/L in sodium citrate and 148.5 ×10^9^/L in lithium heparin. Other hematological parameters show relative stability across the samples, reinforcing that the thrombocytopenia detected in the EDTA tube is likely an artifact rather than a true pathological condition. This observation aligns with the phenomenon known as EDTA-PTCP, which is a rare in vitro occurrence triggered by EDTA-dependent antiplatelet antibodies that cause platelet aggregation, resulting in inaccurately low counts on automated analyzers. It is essential to recognize this condition to prevent unnecessary clinical actions, such as platelet transfusions or extensive hematological evaluations. This case highlights the necessity of conducting repeat tests with alternative anticoagulants for patients presenting with unexpectedly low platelet counts in EDTA samples.

This phenomenon is comparatively rare, yet it remains an essential diagnostic factor in clinical practice, especially when abnormally low platelet counts are detected in EDTA-treated blood samples. While the precise prevalence of EDTA-PTCP may vary across different populations and diagnostic contexts, it is frequently underdiagnosed due to its transient nature and the dependence on automated platelet count analyzers, which can wrongly classify platelet aggregation as an actual decrease in platelet levels. Pseudothrombocytopenia is a clinical concept resulting from the clustering of platelets following cold agglutination or EDTA-platelet interaction [5]. EDTA is a polyprotic acid containing four carboxylic acid groups and two amine groups with lone pair electrons, capable of donating more than one proton, giving it the ability to chelate or complex metal ions. Numerous enzyme processes in the coagulation cascade depend on calcium, and its removal permanently stops blood clotting and stabilizes the blood in a fluid form.

Blood clots are formed rapidly when samples are collected in containers without additives. EDTA has demonstrated the best long-term stability of blood particles and cells. Due to this characteristic, EDTA has become the preferred anticoagulant for hematological tests [6].

In EDTA-PTCP, IgG and IgM class antibodies are commonly implicated in reaction with platelets at room temperature [7], and rarely IgA. While the exact mechanism of the reaction is unclear, it is apparent that the antibody immunoglobulin combining site has a huge role [7]. In EDTA-PTCP, the platelet cell membrane has undergone structural changes, resulting in antibodies binding to genetically mutated antigens known as “neoantigens.” Glycoproteins (GP) IIb or I have been suggested to be the neoantigen in PTCP, as platelets of individuals with Glanzmann thrombasthenia do not interact with these antibodies [7]. Moreover, the antibody binding site of GP IIb is normally obscured in the GP IIb/IIIa heterodimer complex. For antibody binding to take place, the dissociation of the complex must occur. The dimer dissociates when calcium concentration is low; thus, EDTA's calcium-chelating effect causes antigen-antibody binding [8,9]. Other factors, such as pH, temperature, and various drugs, can cause this dissociation [10].

When the antiplatelet antibody identifies the GP IIb/IIIa receptors, it stimulates platelet activation markers such as CD62P (P-selectin), CD63 (Type III lysosomal GP), and thrombospondin, hence triggering the activation of tyrosine kinase pathway leading to platelet clumping and a drastic reduction in platelet count [10-13].

In addition to EDTA-PTCP, elevated WBC count has been observed. This occurs as platelet clumps >35 fL formed in PTCP are miscounted as WBCs by analyzers such as the Beckman Coulter LH 750 (Beckman Coulter, Inc., CA), which classifies cells by size [14]. Factitiously elevated WBC can lead to misdiagnosis and unnecessary investigations and may mask true leukopenia, delaying treatment. A similar phenomenon has been reported in a neonate delivered at 39 weeks to a mother with EDTA-dependent PTCP, though it was transient [15].

Xiao et al. reported that platelet count decreased and clumping increased over time, while WBC count falsely increased. In their study, 92.3% of PTCP cases had elevated WBC, with 72.9% within the normal range and 26.1% showing pseudoleukocytosis [16].

**Figure 1:**
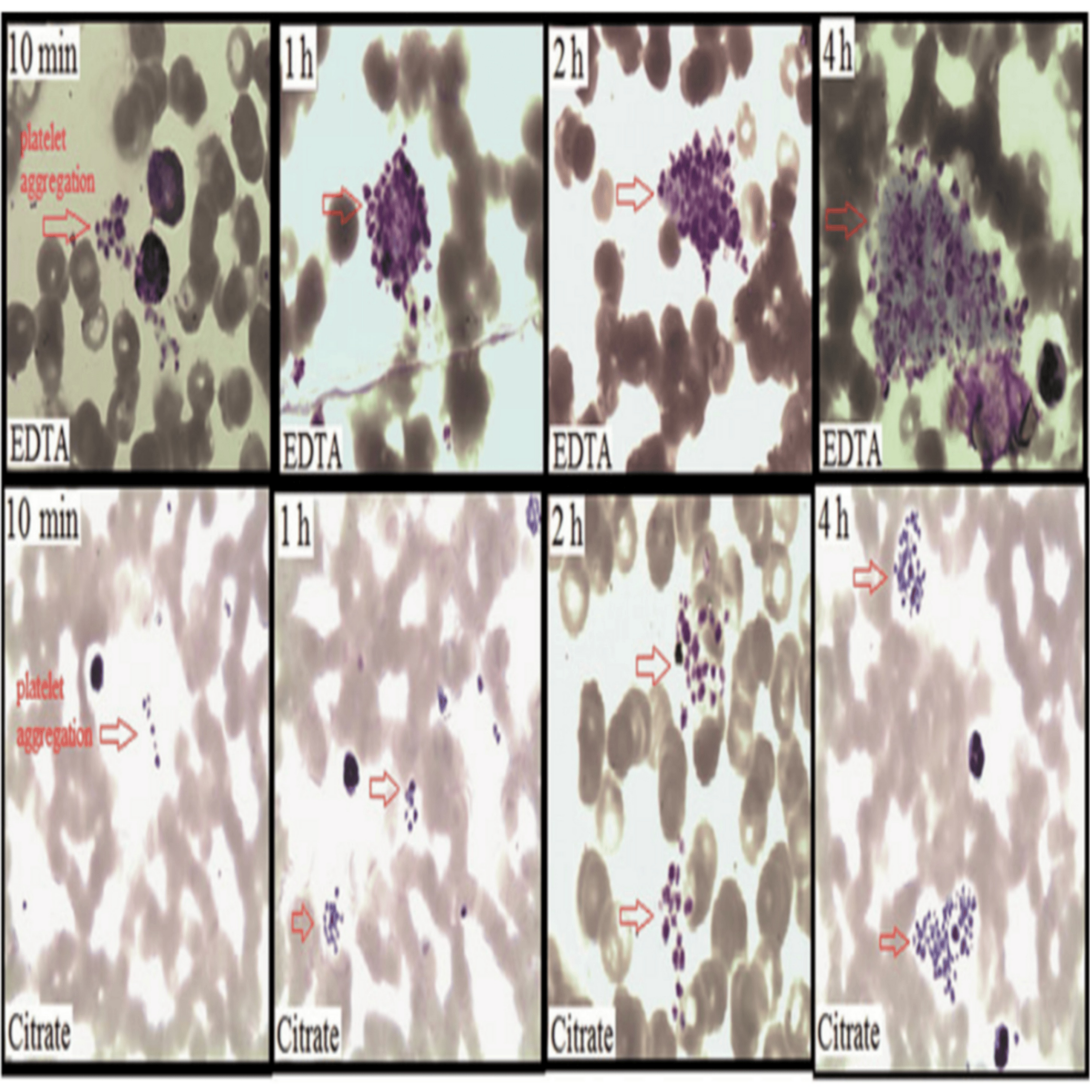
Platelet aggregation of blood smear sample collected in EDTA anticoagulant and citrate anticoagulant at different time points using Wright Stain. EDTA: Ethylene diamine tetra-acetate

Lin et al. [17] analyzed 26 patients with samples in EDTA and citrate tubes. EDTA-PTCP occurred within 10 minutes, with platelet levels declining over two hours. Platelet aggregation was also noted in 17 citrate samples after one hour, with a less marked effect than EDTA (Figure 1).

Several methods have been explored to mitigate EDTA-PTCP, including the use of antiplatelets such as acetylsalicylic acid (ASA), prostaglandin E1 (PGE1), apyrase, or monoclonal antibodies. Other substances used include sodium citrate, heparin, ammonium oxalate [18], β-hydroxyethyl theophylline [19], potassium azide [19], kanamycin, and other aminoglycosides. Warming samples to 37°C has also shown efficacy in reducing clumping.

Chae et al. proposed an innovative method based on the mechanism of EDTA-related PTCP. This involves adding 0.01 mL of calcium chloride (CaCl_2_) and heparin to 0.5 mL of whole blood 30 minutes post-collection, then allowing it to stand for 60 minutes before platelet counting. This method achieved similar results to kanamycin supplementation, showing a 2.4-fold increase in mean platelet count while preserving cell morphology [20].

## Conclusions

In patient care, laboratory investigations are crucial in decision-making and treatment planning. However, errors can arise, particularly during the extra-analytical phase, such as sample collection, storage, and handling, which can negatively impact patient outcomes and the overall quality of care. While EDTA-PTCP may lead to inappropriate interventions, it should not be classified strictly as a laboratory error, but rather as a diagnostic consideration requiring a high index of suspicion. Importantly, it can also reflect a pre-analytical or analytical issue that extends beyond clinical awareness, emphasizing the need for increased laboratory vigilance and communication between clinicians and laboratory personnel. This condition should be considered, especially in patients without a history of bleeding disorders or clinical conditions such as disseminated intravascular coagulation (DIC).
